# Spectrum of Electroencephalography Findings in Newly Diagnosed Epilepsy

**DOI:** 10.7759/cureus.15938

**Published:** 2021-06-26

**Authors:** Muhammad A Khalily, Muhammad Akhtar, Shaila Ali, Shumaila Rafique, Tipu Sultan, Areeba Wasim

**Affiliations:** 1 Department of Pediatric Neurology, The Children's Hospital and The Institute Of Child Health, Lahore, PAK

**Keywords:** spectrum, eeg, epilepsy, discharges, seizures

## Abstract

Background

Epilepsy is a neurological disorder that presents with recurrent seizures associated with erratic brain activity which can be measured through EEG in addition to other neurological investigations. However, EEG may show abnormal patterns and waveforms while the patient is having a seizure which is crucial for making an accurate diagnosis.

Objective

This study aims to evaluate the spectrum of EEG findings in newly diagnosed epileptic patients as part of a neurological investigation.

Material and methods

This cross-sectional study was carried out at the Department of Paediatric Neurology, the Children's Hospital, and the Institute of Child Health, Lahore for six months. A sample of 122 patients was enrolled in this study with an age range of >1 month and <18 years, with a diagnosis of epilepsy based upon ≥2 unprovoked seizures that occurred ≥ 24 hours apart. After obtaining informed consent from the patients, a one-time EEG was carried out and details were noted such as type and frequency of the discharge, site of maximum amplitude, paroxysm morphology, and onset and offset (focal/generalized) of the discharges. The data was analyzed using SPSS v.25 (IBM SPSS Statistics for Windows, Armonk, NY).

Results

The mean age of children enrolled in this study was 5.58 ± 3.46 years. There were 70 (57.4%) males and 52 (42.6%) females. The mean age at the onset of seizures was 4.85 ± 3.16 years. Out of 122 children, focal onset aware epilepsy type was noted in 8 cases, focal onset impaired awareness was noted in 19 cases and generalized onset motor type of epilepsy was noted in 95 cases. Furthermore, EEG findings were normal in 41 (33.61%) patients; however, 81 (66.39%) EEG findings of the patients place them in the abnormal range. On EEG, paroxysm morphology was typical in 78 (96.3%) patients while atypical in 3 (3.7%) patients. Discharge spectrum was generalized in 46 (56.8%) patients, localized in 19 (23.5%) patients, bilateral independent in 1 (1.2%) patient and multifocal in 15 (18.5%) patients. Discharge pattern was periodic in seven (8.6%) cases, rhythmic delta activity was noted in 4 (4.9%) cases, spike and wave pattern was noted in 68 (84.0%) cases and sharp and wave pattern was observed in 36 (44.4%) patients.

Conclusion

Our study concluded that EEG findings were abnormal in 81 (66.39%) patients. Thus to make the recommendations locally and nationally, we observed that EEG can highlight the abnormal pattern and discharges in newly diagnosed individuals with epilepsy. Our findings could be instrumental to identify the type of EEG discharges in a timely fashion while making diagnoses and treatment plan protocols accordingly. This study finding recommends the early application of EEG after the presentation of epileptic symptoms by the patient. We further recommend that further similar studies be conducted in multiple tertiary care settings to reach a firm and valuable conclusion.

## Introduction

Epilepsy is a neurological condition, characterized by frequent seizures and abnormal brain behavior. Epilepsy requires urgent medical attention and, in many cases, long-term treatment. In various world societies, the frequency is about 0.3-0.5 percent, with a prevalence rate of 5-10 per 1000 people [1]. Nevertheless, the prevalence of epilepsy in Pakistan is estimated to be 9.99/1000. Pakistan has the highest prevalence of epilepsy in younger patients aged 30 or less, accounting for around 2 million individuals and 1/10th of the world's epilepsy burden [2].

The primary aim of electroencephalography (EEG) is to identify the exact type of seizure and epilepsy syndrome so that care can be provided accordingly and also detect unexplained paroxysmal spells that could be seizures [3]. The range of EEG readings is determined by the form of epilepsy. The disorder is divided into two classes by the “International Classification of Epileptic Syndromes and Epilepsies”: partial or focal versus systemic, and idiopathic versus cryptogenic or symptomatic [4].

A bilateral, symmetrical, and synchronous spike-wave behavior on a normal basis is the classic EEG irregular reading of hereditary generalized epilepsy. Generalized polyspikes, generalized polyspike-wave discharges, photoparoxysmal response, eye-closure sensitivity, fixation-off sensitivity, and occipital intermittent rhythmic delta activity are other common EEG anomalies identified in the literature [5]. Atypical EEG anomalies in patients with generalized epilepsy have also been documented by several authors. During non-rapid eye movement sleep, these involved focal, unilateral, and asymmetric discharges, generalized paroxysmal quick operation, and spike-wave morphology distortion [6-8].

Due to inconsistency in previous studies, it is hard to generalize the findings of the EEG abnormalities while making a diagnosis of epilepsy. The rationale of this study is to evaluate the wide spectrum of EEG abnormalities in children with epilepsy. This would help to get an estimate of the prevalence of the vast spectrum of EEG findings in epileptic patients; consequently, leading us to identify any atypical EEG findings in patients presenting with seizures which could have otherwise potentially led to inaccurate diagnosis and inappropriate choice of antiepileptic drug therapy. Therefore, we conducted this study to make some recommendations locally and nationally as there are limited relative studies published in Pakistan about newly diagnosed epilepsy cases highlighting abnormal patterns and discharges.

## Materials and methods

This cross-sectional study was carried out at the Department of Paediatric Neurology, the Children's Hospital, and the Institute of Child Health, Lahore for six months (22/07/2020 to 21/01/2021). The sample size was calculated by using Select Statistics sample size calculator (https://select-statistics.co.uk/calculators/) where the confidence level was 95%, and margin of error was 5.5%, prevalence of epilepsy was 9.99 per 1000 population [2]. The sample size was 122 patients, a non-probability, consecutive sampling. The participants enrolled in the study were children of ages greater than one month and less than 18 years, both genders, who presented with first episodes of epilepsy based upon two or more unprovoked seizures occurring more than 24 hours apart, first unprovoked seizures, epileptic patients with normal development and normal neurological examination.

We used the definition of epilepsy according to International League Against Epilepsy (ILAE) as when “at least two or more unprovoked seizures occurring more than 24 hours apart” or “one unprovoked seizure and a probability of further seizures similar to the general recurrence risk (at least 60%) after two unprovoked seizures, occurring over the next 10 years”[9]. Children with focal neurological deficit, epileptic encephalopathies, having abnormal neurocutaneous signs, developmental disorder or degenerative brain disease were excluded from the study. All patients who fulfilled the inclusion criteria were enrolled for the study.

Ethical approval from the institutional ethical review committee (2020-123-CHICH) was attained prior to the study. After obtaining informed consent from the patients, a one-time encephalography (EEG) was conducted. Clinical and demographic information was collected. The gathered information like current age of child, sex of child, type of seizure, age at the time of first episode of seizures and duration of recent seizure. An expert EEG reader reviewed all the EEGs readings. Detailed evaluation of all abnormal epileptiform readings was done. Details of each discharge was noted in the proforma. The details of discharges listed type and frequency of the discharges, site of the maximum amplitude, paroxysm morphology and onset and offset (focal/generalized/multifocal/bilateral independent) and pattern (spikes, spike and wave, sharp and wave, periodic discharges, rhythmic delta activity) of the discharges. The spectrum of EEG findings was defined as "the various epileptiform both typical and atypical patterns that are whether generalized, focal or multifocal and (i) spikes (<70msec duration), (ii) spike and wave, (iii) sharp and wave (70-200ms duration), (iv) rhythmic delta activity, (v) periodic discharges” [3]. The data was entered and analyzed by SPSS v.25 (IBM SPSS Statistics for Windows, Armonk, NY). Mean ± SD was estimated for age and duration of discharge. Frequency and percentage was estimated for sex of child, as well as EEG characteristics like morphology, symmetry, and focal discharge.

## Results

Out of 122 children, focal onset aware epilepsy type was noted in 8 cases, focal onset impaired awareness was noted in 19 cases, and generalized onset motor type of epilepsy was noted in 95 cases. For additional details, see Tables 1-2.

**Table 1:** Baseline features of patients

N	122
Age (years)	5.58 ± 3.46
Gender	
Male	70 (57.4%)
Female	52 (42.6%)
Age at onset of seizures (years)	4.85 ± 3.16
Duration of epilepsy symptoms (months)	4.39 ± 4.62
Time since last seizure (days)	2.29 ± 3.06
Duration of seizures	
<5 min	76 (62.3%)
5-10 min	46 (37.7%)
First afebrile seizure	
Yes	16 (13.1%)
No	106 (86.9%)
Epilepsy in first-degree relative(s)	
Yes	1 (0.8%)
No	121 (99.2%)

**Table 2:** Distribution of type of epilepsy

Epilepsy type	Subtype	F (%)
Focal onset aware	Clonic, myoclonic, tonic clonic	3 (2.4%), 3 (2.4%), 2 (1.6%)
Focal onset impaired awareness	Myoclonic, tonic clonic, tonic with up-rolling eyes	3 (2.4%), 13 (10.6%), 3 (2.4%)
Generalized onset motor	Clonic, clonic with up-rolling eyes, clonic and myoclonic, tonic clonic, myoclonic, tonic with myoclonic	1 (0.8%), 1 (0.8%), 1 (0.8%), 88 (72.1%), 2 (1.6%), 2 (1.6%)
Generalized onset non-motor		0 (0.0%)
Unknown onset or unclassified		0 (0.0%)

EEG was performed within 24 hours of fits in 59 (72.8%) patients, within 24-48 hours in 15 (18.5%) patients, within 48-72 hours in 19 (23.5%) patients and after 72 hours in 29 (35.8%) patients. On EEG, paroxysm morphology was typical in 78 (96.3%) patients while atypical in 3 (3.7%) patients. Discharge spectrum was generalized in 46 (56.8%) patients, localized in 19 (23.5%) patients, bilateral independent in 1 (1.2%) patient and multifocal in 15 (18.5%) patients. Discharge pattern was periodic in 7 (8.6%) cases, rhythmic delta activity was noted in 4 (4.9%) cases, spike and wave pattern was noted in 68 (84.0%) cases and sharp and wave pattern was observed in 36 (44.4%) patients as given in Table 3. 

**Table 3:** Spectrum of epileptic seizures (n = 81)

		Frequency
EEG performed	Within 24 hours	59 (72.8%)
	24-48 hours	15 (18.5%)
	48-72 hours	19 (23.5%)
	>72 hours	29 (35.8%)
Paroxysm morphology	Typical	78 (96.3%)
	Atypical	3 (3.7%)
Discharge spectrum	Generalized	46 (56.8%)
	Localized	19 (23.5%)
	Bilateral independent	1 (1.2%)
	Multifocal	15 (18.5%)
Discharge pattern	Periodic discharges	7 (8.6%)
	Rhythmic delta activity	4 (4.9%)
	Spike and wave pattern	68 (84.0%)
	Sharp and wave pattern	36 (44.4%)

Data was stratified for age and gender of children. In children aged ≤5 years, EEG was abnormal in 39 patients, in children aged 6-9 years, EEG was abnormal in 32 patients and in children aged ≥10 years, EEG was abnormal in 10 patients. The difference was insignificant (p>0.05). In male children, EEG was abnormal in 49 patients while in females, EEG was abnormal in 32 patients. The difference was insignificant (p>0.05) in both genders as given in Table 4.

**Table 4:** Comparison of ECG findings in genders and different age strata

	EEG findings		p-value
	Normal	Abnormal	
Age ≤5 years	22	39	0.666
Age 6-9 years	16	32	
Age ≥10 years	3	10	
Male	21	49	0.328
Female	20	32	

**Figure 1:**
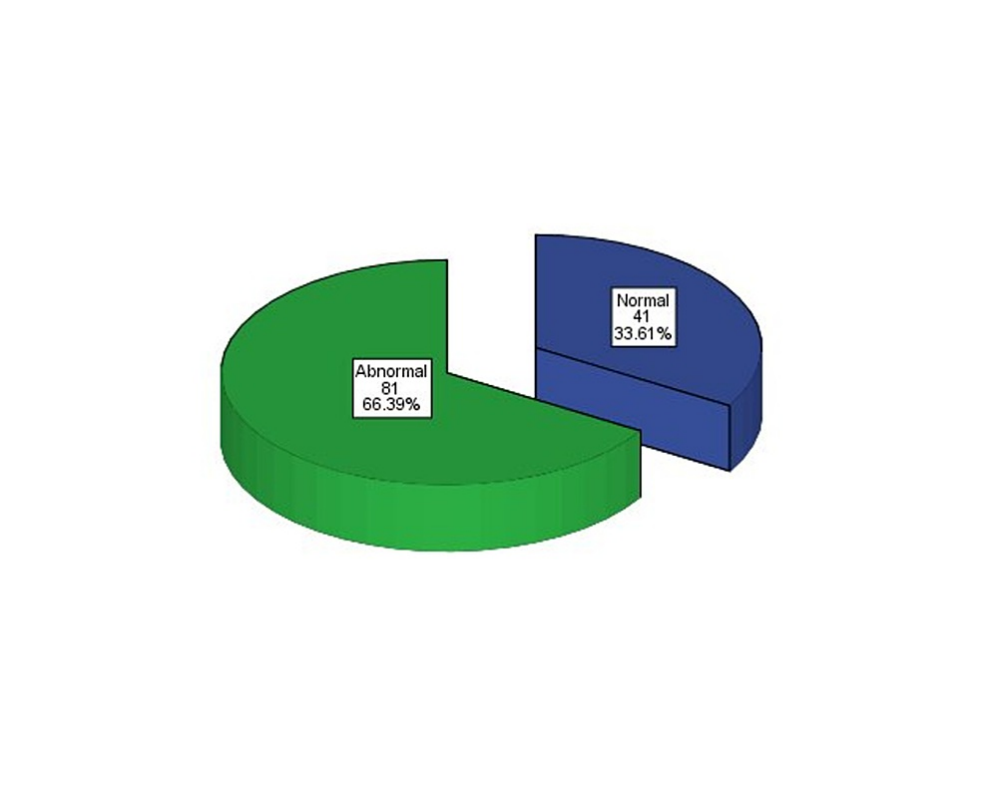
EEG findings

Out of 122 children, EEG was overall normal in 41 (33.61%) patients, while EEG was abnormal in 81 (66.39%) patients as given in Figure 1.

## Discussion

Apart from clinical history and neurological examination, an EEG is a common investigation used in the diagnosis of epilepsy. The EEG is the recording of the electrical impulses produced by the nerve cells in the brain, and it is a very useful therapeutic instrument for epilepsy diagnosis and treatment [10]. To diagnose epilepsy, a continuous recording of the EEG for up to one week is needed. Electrodes are attached to the patient's scalp for this test. The electrodes capture the brain's electrical activity. An ambulatory EEG is also available, which the patient can wear at home for a few days while the EEG tracks seizure activity [11].

It is usual for epileptic patients to have normal EEG readings. However, EEG may show abnormal patterns and waveforms while the patient is having a seizure (interictal EEG) which is crucial for making an accurate diagnosis [12]. These findings may be diverse depending upon the type and site of abnormal impulse discharge. It may include non-epileptiform abnormalities and interictal epileptiform discharges. This is the most important diagnostic finding that is consistent with the diagnosis of epilepsy. To prevent overinterpreting benign variants and artifacts that could be mistaken for epileptiform behavior, interictal epileptiform discharges must be closely differentiated from benign variants or regular brain waves [13].

In our study, the mean age of children enrolled in this study was 5.58 ± 3.46 years. There were 70 (57.4%) males and 52 (42.6%) females. The mean age at the onset of seizures was 4.85 ± 3.16 years. Out of 122 children, focal onset aware epilepsy type was noted in eight cases, focal onset impaired awareness was noted in 19 cases and generalized onset motor type of epilepsy was noted in 95 cases. EEG was overall normal in 41 (33.61%) patients, while abnormal in 81 (66.39%) patients. On EEG, paroxysm morphology was typical in 78 (96.3%) patients while atypical in three (3.7%) patients. Discharge spectrum was generalized in 46 (56.8%) patients, localized in 19 (23.5%) patients, bilateral independent in one (1.2%) patient and multifocal in 15 (18.5%) patients. Discharge pattern was periodic in seven (8.6%) cases, rhythmic delta activity was noted in four (4.9%) cases, spike and wave pattern was noted in 68 (84.0%) cases and sharp and wave pattern was observed in 36 (44.4%) patients.

Rajper et al. conducted a study in Karachi and found that generalized seizures were observed in 86.7% of cases while 13.3% of cases had focal seizures. In about 45.3% of cases, the EEG was done within the first 24 hours of presentation, which was almost similar as we observed in our study (48.4%). They further observed that EEG was abnormal in 55.2% cases, while in our study, EEG was abnormal in 66.39% cases. A history of abnormalities was observed in 15.6% of patients, interictal epilepticus discharges in 20.9%, and interictal epilepticus discharges with background slowing in 18.7% [14].

However, in another study, Samra et al. found that the frequency of epileptiform EEG abnormality was 40% in all children presenting with seizures, and the overall frequency of confirmed epilepsy was about 30%. Thus EEG abnormality was 13.3% associated with the diagnosis of epilepsy [15]. Another study, done by King et al., showed that 51% of patients had interictal epilepticus discharges, whose EEG was done with 24 hours of seizures [16].

Interictal discharges were seen in the first EEG in about 18-56% of patients with new-onset epilepsy, but when a second sleep-dependent EEG was conducted, the percentage increased to 61%, according to a report by Wirrell et al. [17] EEG has a precision of 78-98%, which is higher than the sensitivity of 25-56% [18].

The presence of epileptiform EEGs also in the absence of epilepsy has recently piqued researchers' attention. Some researchers believe that these anomalies may play a causal role in the autism phenotype, claiming that rates as high as 60% have been registered. The care effects are becoming extremely significant, considering the fact that this syndrome is still not fully known and risk factors are yet to be determined [19].

Hypsarrhythmia in infantile spasm, burst suppression in early infantile epileptic encephalopathy, generalized 3 Hz pulse wave discharges in absence seizures, and intermittent complexes in subacute sclerosing panencephalitis are all common classic epileptiform patterns seen in infancy [20]. For the diagnosis of seizure, epilepsy form, and epileptic syndrome of children that have experienced their first unprovoked seizure, EEG is prescribed as a first-tier inquiry. It may be helpful in predicting the long-term result or recurrence of a condition [21].

When compared to children with regular EEG, people with focal epileptiform discharges have a greater chance of recurrence [22]. These history anomalies are often temporary, so an EEG can be replicated for a certain amount of time to see if the abnormality persists [23].

## Conclusions

In our study, we conclude that EEG findings were abnormal in 81 (66.39%) patients. Thus to make the recommendations locally and nationally, we observed that EEG can highlight the abnormal pattern and discharges in newly diagnosed individuals with epilepsy. Our findings could be instrumental to identify the type of EEG discharges in a timely manner while making appropriate diagnoses and treatment plan protocols accordingly. The findings of this study recommend the early application of EEG after the presentation of epileptic symptoms by the patient. We further recommend that further similar studies be conducted in multiple tertiary care settings to reach a firm and valuable conclusion. 
